# Myocardial Revascularization in Patients with Diabetes and Heart Failure—A Narrative Review

**DOI:** 10.3390/ijms26073398

**Published:** 2025-04-05

**Authors:** Stefan Zivkovic, Aleksandar Mandic, Kosta Krupnikovic, Aleksa Obradovic, Vojko Misevic, Mihajlo Farkic, Ivan Ilic, Milorad Tesic, Srdjan Aleksandric, Stefan Juricic, Branko Beleslin, Milan Dobric

**Affiliations:** 1 Cardiology Clinic, Institute for Cardiovascular Diseases “Dedinje”, 11000 Belgrade, Serbia; aleksandar.mandic1993@gmail.com (A.M.); krupnikovick@gmail.com (K.K.); obradovicaleksa96@gmail.com (A.O.); misevic.vojko@gmail.com (V.M.); mfarhi@gmail.com (M.F.); ivan1ilic@yahoo.com (I.I.); 2 Faculty of Medicine, University of Belgrade, 11000 Belgrade, Serbia; misa.tesic@gmail.com (M.T.); srdjanaleksandric@gmail.com (S.A.); branko.beleslin@gmail.com (B.B.); 3 Cardiology Clinic, University Clinical Center of Serbia, 11000 Belgrade, Serbia; stefan.juricic@gmail.com

**Keywords:** diabetes mellitus, heart failure, coronary artery disease, myocardial revascularization

## Abstract

Heart failure and diabetes mellitus are major contributors to global morbidity and mortality, with their prevalence continuously rising, primarily due to aging populations and improvements in healthcare. These conditions often coexist or develop sequentially, leading to complex interactions that significantly influence the progression and management of both diseases. Furthermore, heart failure and diabetes are commonly associated with coronary artery disease, which presents a unique challenge in clinical management, particularly in the context of myocardial revascularization. The presence of diabetes exacerbates atherosclerotic progression and impairs endothelial function, while heart failure complicates the perfusion and recovery of myocardial tissue post-intervention. This narrative review delves into the underlying mechanisms contributing to revascularization failure in patients with heart failure and diabetes, emphasizing the importance of understanding these interactions for optimal treatment. The review also summarizes key findings from randomized controlled trials, examining evidence both in the general population and in specific subgroups, including the elderly and patients with left main coronary artery disease, chronic kidney disease, peripheral artery disease, and chronic obstructive pulmonary disease. Understanding these complexities is critical for improving patient outcomes.

## 1. Heart Failure: Definition, Epidemiology, and Types

Heart failure is a clinical syndrome characterized by the heart’s inability to pump blood effectively to meet the body’s metabolic demands, or meeting this requirement only at elevated filling pressures. This condition typically results from structural or functional impairments of the heart muscle and is often associated with reduced cardiac output [1,2].

Heart failure represents a significant global public health issue and is one of the leading causes of morbidity and mortality, particularly among the elderly. The estimated prevalence in adults ranges from 1% to 2%, reaching approximately 10% in individuals aged over 65 years. The prevalence continues to rise, primarily driven by the aging population, improved survival following myocardial infarction, and better management of other cardiovascular diseases. Despite advancements in treatment, mortality rates remain high, with approximately 50% of individuals diagnosed with heart failure dying within five years of diagnosis. Prognosis, however, varies significantly depending on the type of heart failure, comorbidities, and the quality of clinical management [3].

Heart failure can be classified based on several criteria, including the affected ventricle (left-sided, right-sided, or both-sided), the left ventricular global systolic function (heart failure with reduced ejection fraction [HFrEF], with a left ventricular ejection fraction (LVEF) ≤ 40%, previously referred to as “systolic heart failure”; heart failure with mildly reduced ejection fraction [HFmrEF], with an LVEF of 41–49%; and heart failure with preserved ejection fraction [HFpEF], with an LVEF ≥ 50%, previously termed “diastolic heart failure”), and the clinical presentation (acute or chronic) [2]. Ischemic heart failure, a subtype primarily caused by coronary artery disease (CAD), results from a prior myocardial infarction or chronic myocardial ischemia. It is one of the most prevalent forms of heart failure, especially in developed countries [4]. This article focuses on chronic left-sided ischemic heart failure with reduced ejection fraction.

## 2. Pathophysiological and Molecular Mechanisms of Ischemic Heart Failure

There are two types of myocardial ischemia: acute and chronic. These conditions differ in terms of duration and underlying causes of the reduced blood flow through the coronary arteries, resulting in inadequate oxygen supply to the heart muscle. Both conditions can ultimately lead to heart failure.

Acute ischemia occurs suddenly, typically due to a blockage in a coronary artery, often caused by a blood clot or rupture of an atherosclerotic plaque. This blockage leads to a rapid reduction or complete cessation of blood flow, which can result in a myocardial infarction. If not treated promptly, it can lead to ischemic cardiomyopathy. Acute ischemia can also contribute to the development of acute heart failure [5].

Chronic ischemia develops over time, typically due to atherosclerosis, causing gradual narrowing of the coronary arteries. This reduces blood flow and can eventually lead to myocardial injury, weakness, and chronic heart failure [6].

The pathophysiological mechanisms underlying ischemia-induced myocardial injury leading to heart failure are multifactorial. Coronary artery stenosis or occlusion reduces myocardial blood flow, causing hypoxia in the myocardial tissue. The lack of oxygen and nutrients triggers cellular stress, activating several important signaling pathways that can result in cellular dysfunction or death. Oxygen deprivation shifts metabolism to anaerobic pathways, resulting in mitochondrial injury and dysfunction, reduced ATP production, and impaired cellular functions, including diminished Na+/K+ ATPase activity. This dysfunction causes ionic disbalance and cellular swelling. In addition, the sarcoplasmic reticulum ATPase (SERCA) pump becomes impaired, leading to intracellular calcium overload, which contributes to contractile dysfunction and arrhythmias [7]. Anaerobic metabolism also leads to lactate production, which lowers intracellular pH and creates acidotic conditions that further interfere with cellular processes [8].

In ischemic heart failure, members of the TGF-β (transforming growth factor beta) superfamily play a key role in regulating inflammatory, reparative, fibrogenic, and hypertrophic processes through Smad-dependent and Smad-independent pathways. Smad 1 activation in cardiomyocytes offers protective, anti-apoptotic effects during ischemia and reperfusion. Smad 3, on the other hand, contributes to the repair of infarcted cardiac tissue by activating reparative myofibroblasts and promoting an anti-inflammatory phenotype in macrophages. However, prolonged Smad 3 activation can lead to adverse remodeling and fibrosis. The inhibitory Smads, such as Smad 7, modulate TGF-β-driven fibroblast activation while also independently inhibiting receptor tyrosine kinase signaling [9].

Oxidative stress is a critical factor in myocardial infarction and reperfusion injury. Reperfusion following ischemia triggers excessive production of superoxide anions, which react with nitric oxide (NO) to form peroxynitrite (ONOO-). This reactive species causes dysfunction of calcium pumps, vascular endothelial damage, and apoptotic cell death [10].

Increased oxidative stress also intensifies endoplasmic reticulum (ER) stress, which plays a pivotal role in heart failure. Histological evidence of ER hyperplasia in heart failure suggests ER overload. ER stress is prominent in chronic myocardial ischemia-induced heart failure and in models of myocardial overload caused by stress, with ER stress-associated apoptosis contributing significantly to the progression of heart failure in these conditions [11].

Moreover, myocardial ischemia induces the generation of reactive oxygen species (ROS) as a byproduct of mitochondrial dysfunction and cellular stress. Excess ROS contributes to further tissue damage by oxidizing lipids, proteins, and DNA, thus triggering cell apoptosis and necrosis, which results in the loss of viable myocardial tissue. These processes activate signaling pathways that exacerbate inflammation and fibrosis within the myocardium. In response to ischemic injury, inflammatory pathways are activated, including the release of pro-inflammatory cytokines such as TNF-α (tumor necrosis factor-alpha), IL-1β (interleukin-1beta), and IL-6 (interleukin-6), as well as the recruitment of inflammatory cells to the ischemic region. This cascade amplifies myocardial injury and contributes to fibrosis and ventricular remodeling. Ischemia-induced cell death, apoptosis, or necrosis triggers fibroblast activation and deposition of extracellular matrix proteins, primarily collagen. This collagen deposition results in the formation of fibrous tissue, ventricular remodeling, and a reduction in myocardial contractility, contributing to the progression of heart failure [11,12].

Several genetic and molecular mechanisms contribute to pathological remodeling, including the hypertrophic response mediated by key signaling pathways such as MAPK (mitogen-activated protein kinase), calcineurin/NFAT, and PI3K/Akt. These pathways induce adaptive hypertrophy to maintain cardiac output. While compensatory hypertrophy initially supports heart function, it eventually leads to pathological remodeling and worsens the progression of heart failure. In addition, the activation of the TGF-β pathway in ischemic heart failure promotes fibroblast recruitment and collagen deposition, further contributing to cardiac fibrosis [13,14].

To counterbalance the diminished cardiac output, the body initiates various homeostatic mechanisms, such as the activation of the sympathetic nervous system and renal compensation via the renin–angiotensin–aldosterone system (RAAS). However, in advanced stages of heart failure, these compensatory mechanisms become insufficient, and cardiac output declines critically [15].

## 3. Diabetes Mellitus: Definition, Types, and Epidemiology

Diabetes mellitus is a chronic metabolic disorder characterized by persistent hyperglycemia, resulting from either insulin resistance or impaired insulin secretion. The two most common forms of diabetes are Type 1 diabetes (T1DM), previously known as insulin-dependent or juvenile-onset diabetes, which typically manifests in childhood and accounts for approximately 10% of cases, and Type 2 diabetes (T2DM), previously referred to as insulin-independent or adult-onset diabetes, which is the more prevalent form, representing about 90% of cases. Both forms are associated with various complications, including ischemic heart disease and heart failure [16].

In 2021, an estimated 537 million adults aged 20–79 years were living with this disease worldwide. Projections suggest that by 2030, the number of individuals affected will increase to 643 million, and by 2045, to 783 million. It is noteworthy that three out of four cases will occur in low- and middle-income countries [17].

Individuals with diabetes have a two- to fourfold higher risk of developing CAD compared to those without diabetes. Notably, 75% of diabetic patients die from cardiovascular-related causes. Diabetes is a prevalent comorbidity in patients undergoing myocardial revascularization, with 25% to 30% of all percutaneous and surgical procedures being performed in individuals with diabetes. Having diabetes is considered equivalent to having CAD, and diabetic patients without a previous CAD diagnosis have a five-year cardiovascular mortality rate similar to that of nondiabetic individuals with a history of myocardial infarction [18,19]. Furthermore, diabetic patients tend to experience more complex forms of CAD, and after an initial procedure, they face an increased risk of target vessel failure and the need for repeated revascularizations [20].

## 4. Heart Failure and Diabetes Mellitus

Diabetes is commonly observed in patients with heart failure. The coexistence of these two conditions significantly increases mortality risk compared to each condition alone. The mechanisms underlying this increased risk are multifactorial, involving metabolic disturbances that negatively affect the cardiovascular system and myocardial function. Moreover, patients with diabetes are at a substantially increased risk of developing heart failure, with the risk being more than twofold higher in men and more than fivefold in women. Several factors contribute to this heightened risk including age, disease duration, insulin use, CAD, and renal dysfunction [21].

Moreover, many risk factors for heart failure and diabetes overlap, as both conditions are influenced by genetics, lifestyle, and chronic health issues. Common risk factors include age, genetics, obesity, hypertension, physical inactivity, poor diet, and smoking. Overweight and obesity increase the risk of both conditions by promoting insulin resistance, a key factor in diabetes, and contributing to heart failure. Hypertension damages blood vessels and increases the heart’s workload, leading to heart failure. Physical inactivity raises the risk of obesity, hypertension, and insulin resistance, worsening both conditions. A poor diet, high in unhealthy fats, sugars, and processed foods, further contributes to the development of heart failure and diabetes. Although both conditions can develop at any age, the risk rises significantly after age 50. Individuals with a family history of diabetes or heart failure are also at greater risk. Smoking worsens insulin resistance and damages blood vessels, further impairing heart function [22].The progression to heart failure in diabetes involves a complex interaction between metabolic abnormalities and structural changes. These alterations include not only the sequelae of CAD but also the direct effects of disrupted glucose and lipid metabolism on cardiac function [23].

In diabetic individuals, myocardial efficiency in glucose metabolism is compromised. A reduction in glucose transport into the myocardium occurs due to decreased levels of glucose transporter proteins (GLUT1 and GLUT4), impairing both glycolysis and mitochondrial pyruvate oxidation. This impairment reduces the heart’s ability to efficiently produce energy. As a result, there is a shift towards increased oxidation of free fatty acids (FFAs) to generate energy. While initially compensatory, this shift becomes maladaptive over time, leading to reduced cardiac efficiency. Beta-oxidation of FFAs is less efficient in energy production compared to glucose oxidation, resulting in increased oxygen consumption and making the heart more vulnerable to dysfunction, particularly during periods of increased metabolic demand or ischemia [24]. These metabolic changes in diabetes contribute to both systolic and diastolic left ventricular dysfunction, even in the absence of overt CAD [25]. Diabetes accelerates myocardial fibrosis and increased collagen deposition, which can increase myocardial stiffness and further impair ventricular relaxation. In addition, altered intracellular calcium handling, due to insulin resistance and disrupted glucose metabolism, exacerbates contractile dysfunction. Hyperglycemia, insulin resistance, and elevated FFAs significantly contribute to oxidative stress, which damages myocardial cells and worsens heart failure. These factors also activate the local RAAS and the sympathetic nervous system, promoting vasoconstriction, fluid retention, and increased cardiac workload, which further impair myocardial function [25,26].

## 5. Diabetes Mellitus, Atherosclerosis, and Revascularization Failure

**Figure 1:**
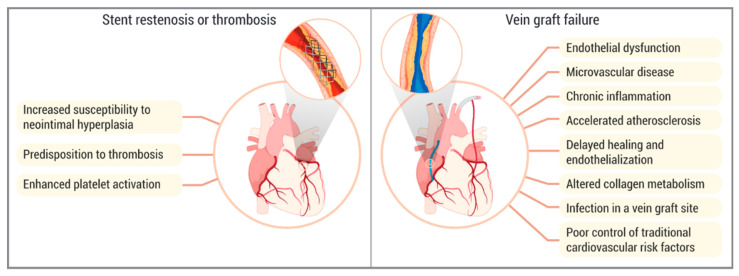
Diabetes’ contribution to myocardial revascularization failure.

Diabetes plays a significant role in the development and progression of atherosclerosis, restenosis, stent thrombosis, and graft failure, through a range of pathophysiological mechanisms (Figure 1). These mechanisms primarily involve alteration in inflammatory pathways due to hyperglycemia, insulin resistance, and changes in FFA metabolism. As a result, diabetic patients are predisposed to endothelial dysfunction, thrombogenesis, monocyte activation, foam cell transformation, and altered smooth muscle cell migration, all of which contribute to the increased coronary artery plaque burden and more complex CAD [18].

### 5.1. Endothelial Dysfunction and Inflammation

The endothelium plays a crucial role in maintaining vascular tone and regulating blood flow. Disruption of endothelial cell function increases the activity of smooth muscle cells, leukocytes, and platelets, and promotes atherosclerosis. In healthy individuals, insulin stimulates the phosphoinositol-3 kinase signaling pathway in endothelial cells, leading to the production of NO, a key molecule that promotes vasodilation and exerts antiplatelet, antiproliferative, and antioxidant effects [27]. However, in patients with T2DM, insulin resistance impairs NO production, resulting in diminished vasodilation and endothelial dysfunction [27,28].

Furthermore, diabetes is associated with increased levels of pro-inflammatory cytokines, such as TNF-α and IL-6. These cytokines bind to endothelial receptors and activate NF-κB (nuclear factor-kappa B), which promotes the transcription of endothelial adhesion molecules. Increased expression of these molecules facilitates the binding of leukocytes and platelets to the endothelium, enhancing thrombogenesis and contributing to plaque instability, which accelerates the progression of CAD [29].

### 5.2. Platelet Activation and Advanced Glycation End Products

In addition to endothelial dysfunction, platelet activity is heightened in diabetic patients. This is due to the increased expression of P-selectin on platelet surfaces and the glycation of platelet receptors, which enhances platelet adhesion. Diabetes is also characterized by increased levels of advanced glycation end products (AGEs), formed when reduced carbohydrates such as glucose bind to free amino groups in proteins, lipids, and nucleic acids. AGEs activate receptors for AGEs (RAGEs) on endothelial cells, triggering the production of pro-inflammatory cytokines and growth factors, and disrupting NO production [30]. These processes contribute to vascular dysfunction, tissue proliferation, and extracellular matrix remodeling. Moreover, the AGEs are implicated in various vascular complications in diabetes, including accelerated atherosclerosis and thrombosis [31].

### 5.3. Neointimal Hyperplasia and In-Stent Restenosis

Patients with diabetes are more susceptible to developing neointimal hyperplasia, which leads to in-stent restenosis. This is partly due to the increased production of TGF-β (transforming growth factor-beta) and enhanced migration and proliferation of smooth muscle cells in response to the hyperglycemic environment. Studies have shown that accelerated neointimal hyperplasia occurs more frequently in diabetic patients after angioplasty [32].

### 5.4. Stent Thrombosis

The neointima formed after stenting in diabetic patients exhibits biological characteristics making it prone to thrombosis. Optical coherence tomography reveals a low-signal pattern in the neointima, thought to be associated with the increased proteoglycans content and organized thrombi [33]. Platelets in diabetic patients are more reactive than those in nondiabetic patients, further increasing the risk of stent thrombosis. Stent thrombosis is a serious complication of coronary intervention and is more common in diabetic patients due to enhanced platelet activation and a heightened tendency for clot formation [34].

### 5.5. Vein Graft Failure

The use of veins for grafting is suboptimal as a long-term substitute for arteries. Over time, the vein undergoes “arterialization”, but this process is often incomplete in diabetic patients. Inadequate adaptation of the vein to the arterial environment leads to graft failure [35]. Several factors contribute to this, including 1. endothelial dysfunction, which is particularly relevant in vein grafts, as they depend on a healthy endothelium for proper function and patency [36]; 2. microvascular disease affecting small blood vessels in grafted veins, which leads to reduced blood flow and poor graft healing [37]; 3. chronic inflammation in the graft and surrounding tissue promoting intimal hyperplasia, which contributes to graft stenosis and occlusion [36,38]; 4. accelerated atherosclerosis [39,40]; 5. delayed healing and endothelialization, combined with platelet dysfunction and a hypercoagulable state which increase the risk of thrombosis and occlusion in the early postoperative period [41]; 6. altered collagen metabolism resulting in abnormal collagen formation, which weakens the graft and increases the risk of its failure [37]; 7. oxidative stress, which worsens endothelial dysfunction, promotes inflammation, and accelerates atherosclerosis in vein grafts, contributing to graft failure [42]; 8. hyperglycemia causing non-enzymatic glycation of proteins and formation of AGEs, impairing the function of collagen, elastin, and other extracellular matrix proteins in vein grafts, and resulting in premature failure [40]; 9. infection at the graft site, which disrupts the healing process, promotes thrombosis, and induces inflammation [43]; and 10. poor control of cardiovascular risk factors such as dyslipidemia and hypertension, further increasing the likelihood of graft failure [38].

## 6. The Aim

The aim of this narrative review was to summarize the most relevant evidence from randomized clinical trials (RCTs) and meta-analyses investigating myocardial revascularization in patients with HFrEF and diabetes, as well as in specific populations, including those with advanced age, left main CAD (LMCAD), chronic kidney disease (CKD), peripheral artery disease (PAD), and chronic obstructive pulmonary disease (COPD).

## 7. Key Clinical Trials

Comparisons of coronary artery bypass grafting (CABG) and percutaneous coronary intervention (PCI), both inter-relatively and to optimal medical therapy (OMT), have been the focus of numerous clinical trials over the years [44,45]. Recent findings in the field highlight the ongoing need for re-evaluation, updates in clinical practice, and identifying the most suitable approaches for distinct patient subgroups [46,47]. This review analyzes data on the efficacy and safety of CABG and PCI in patients with diabetes and heart failure (Table 1). Although these populations are common in clinical practice, RCT evidence remains limited.

The landmark STICH (Surgical Treatment for Ischemic Heart Failure) trial aimed to assess whether outcomes differ in patients with concomitant severe heart failure (LVEF ≤ 35%) and CAD treated with CABG in addition to OMT, compared to OMT alone [48]. A total of 1212 patients were enrolled in the study, approximately 40% of whom had diabetes [49]. Diabetic patients more frequently presented with multivessel CAD, higher LVEF, and smaller left ventricular volumes [50]. The primary outcome—death from any cause—did not show a significant difference between the compared groups after five years of follow-up [49]. Furthermore, no significant difference was observed in the subpopulation of patients with diabetes [50]. However, the ten-year report of the STICHES (STICH Extension Study) trial demonstrated a significantly lower rate of the primary outcome in the CABG group [51].

The second key trial investigating the benefits of myocardial revascularization in heart failure, the REVIVED-BCIS2 (Revascularization for Ischemic Ventricular Dysfunction) trial, evaluated whether additional PCI could improve survival and LVEF in patients with severe heart failure (LVEF ≤ 35%), compared to OMT alone [52]. The study recruited 700 participants, with 39% of those in the PCI group and 43% in the OMT group having diabetes. The main findings indicated that PCI did not significantly impact the primary endpoint defined as death from any cause or hospitalization for heart failure or the key secondary endpoint—LVEF improvement—after two years in the whole study population, and particularly in the subpopulation with diabetes. The only observed benefit was improved quality of life (QOL) at six months. However, at subsequent follow-ups, QOL was reported to be similar between compared groups [53].

The pivotal trial that assessed different myocardial revascularization strategies in diabetic patients, the FREEDOM (Future Revascularization Evaluation in Patients with Diabetes Mellitus: Optimal Management of Multivessel Disease) trial, compared CABG and PCI in patients with diabetes and multivessel CAD [54]. Altogether, 1900 patients participated in the study, but only 1.7% in the CABG group and 3.3% in the PCI group had severe left ventricular dysfunction (LVEF < 40%). A significant reduction in the primary outcome, defined as the composite of all-cause mortality, myocardial infarction, and stroke, was observed in the CABG group. The separation of the survival curves began after the second year and was maintained until the fifth year. This study also reported a significant decrease in all-cause mortality, myocardial infarction, and repeat revascularization separately, as well as an increase in stroke in the CABG group compared with the PCI group [55]. However, the small number of heart failure patients precluded any meaningful conclusions in this subgroup. The results of this trial have had a significant impact on current clinical practice favoring CABG over PCI in diabetic patients with multivessel CAD [56,57,58,59].

Data from subsequent non-randomized trials largely confirm the conclusions of the FREEDOM trial. One notable study by Ramanathan et al. was a population-based retrospective cohort study involving diabetic patients with multivessel CAD and either acute coronary syndrome (ACS) or stable ischemic heart disease (SIHD) undergoing myocardial revascularization. A total of 4661 patients were enrolled, and 4819 myocardial revascularization procedures were performed—2888 PCI and 1932 CABG. As in the FREEDOM trial, only a small proportion of patients had severely impaired left ventricular function. The study demonstrated significantly lower rates of major adverse cardiac and cerebrovascular events (MACCEs), including all-cause mortality, nonfatal myocardial infarction, and nonfatal stroke, in the CABG group. These benefits were observed both in the early (0–30 days) and late (31 days to five years) phases after revascularization, across both the general population and the subpopulations with ACS and SIHD [60].

The CARDia (Coronary Artery Revascularization in Diabetes) trial, another important trial evaluating CABG and PCI in patients with diabetes and multivessel or complex single-vessel CAD, randomized 510 participants. In the CABG group, 15.1% had moderate and 1.3% severe left ventricular dysfunction, while 19.2% had moderate and 1.1% severe dysfunction in the PCI group. No significant difference was found in the primary endpoint, which included death from any cause, myocardial infarction, and stroke after one year of follow-up [61,62]. The CARDia trial did not specifically consider the subgroup of patients with heart failure.

Several key clinical trials have compared various myocardial revascularization strategies—either among themselves or against OMT—in patients with ischemic heart disease, regardless of diabetes status or heart failure. One of the earliest studies, the BARI (Bypass Angioplasty Revascularization Investigation) trial, published in 1996, evaluated CABG against the then-standard PCI, balloon angioplasty, in patients with multivessel CAD [63]. The trial included 1829 participants, approximately 25% of whom had diabetes, with around 80% receiving medical treatment for this condition. After five years, the results revealed no significant difference in all-cause mortality and Q-wave-myocardial-infarction-free survival rate between the observed groups. However, the CABG group exhibited a significantly lower rate of repeat revascularization. Furthermore, in the subgroup of patients with treated diabetes, all-cause mortality was significantly lower in the CABG group compared to the PCI group [64]. The authors did not specifically analyze the outcomes of patients with left ventricular dysfunction. Importantly, the findings regarding all-cause mortality, both in the overall cohort and in the subgroup with treated diabetes, despite opposing trends, were consistent after ten years of follow-up [65].

The SYNTAX (Synergy Between Percutaneous Coronary Intervention with Taxus and Cardiac Surgery) trial compared CABG with PCI in patients with ischemic heart disease and left main and/or three-vessel coronary artery disease (CAD) [66]. The study also evaluated outcomes regarding CAD complexity using the SYNTAX score, a system specifically designed to assess the anatomical characteristics of the disease [67]. A total of 1800 patients were enrolled, with approximately 25% having diabetes and fewer than 2.5% having severely reduced LVEF (LVEF < 30%). The primary outcome was MACCEs, which included death from any cause, stroke, myocardial infarction, and repeat revascularization. Follow-up data at one, three, five, and ten years consistently demonstrated a significantly higher rate of MACCEs in the PCI group than the CABG group, primarily due to higher incidences of myocardial infarction and repeat revascularization. However, all-cause mortality was similar in the observed groups [68,69,70,71]. In addition, there was no difference in ten-year mortality rates independent of diabetes status [72]. The trial was underpowered to draw conclusions for the patients with heart failure.

The COURAGE (Clinical Outcomes Utilizing Revascularization and Aggressive Drug Evaluation) and ISCHEMIA (International Study of Comparative Health Effectiveness with Medical and Invasive Approaches) trials are two major RCTs that evaluated the impact of myocardial revascularization in addition to OMT versus OMT alone in patients with stable CAD and documented myocardial ischemia. The COURAGE trial randomized 2287 patients to receive either PCI plus OMT or OMT alone, while the ISCHEMIA trial randomized 5179 participants to receive either myocardial revascularization (via CABG or PCI) plus OMT or OMT alone. Both trials included a substantial proportion of patients with diabetes (around 35% in COURAGE, and more than 40% in ISCHEMIA). However, only a small percentage had heart failures and severe left ventricular dysfunction, which was an exclusion criterion in these trials. The primary outcomes were a composite of death from any cause and nonfatal myocardial infarction in the COURAGE trial, and a composite of death from cardiovascular causes, myocardial infarction, and hospitalization for unstable angina, heart failure, or resuscitated cardiac arrest in the ISCHEMIA trial. Both trials demonstrated no significant difference in the primary outcomes between compared groups and these findings were consistent regardless of diabetes status [73,74].

In recent years, numerous meta-analyses have been conducted in this field. A notable study by Ahmed et al. included 18 RCTs and nine propensity-matched observational studies, comprising 28,846 patients. This meta-analysis compared CABG and PCI in patients with diabetes. The results demonstrated that PCI was associated with higher rates of all-cause mortality, cardiac mortality, myocardial infarction, MACCEs, and repeat revascularization compared to CABG [75]. Another significant meta-analysis by al-Sadawi et al., involving 23 studies and 10,110 patients with ischemic heart failure, found lower all-cause and cardiovascular mortality rates in the myocardial revascularization group compared to the OMT group. Moreover, these findings were consistent regardless of the revascularization strategy utilized [76]. A further important meta-analysis by Peia et al., which assessed both short- and long-term outcomes of myocardial revascularization in patients with severely reduced LVEF, included 18 studies and 11,686 patients. This analysis revealed a reduction in overall and cardiovascular mortality, as well as in rates of myocardial infarction and repeat revascularization in the CABG group compared to PCI, with no significant difference in stroke incidence between the two groups [77].

Numerous studies have compared the outcomes of CABG and PCI in patients with diabetes. The findings consistently indicate that CABG is associated with superior long-term survival, a lower incidence of myocardial infarction, and a reduced requirement for repeat revascularization. However, the evidence regarding the comparative risk of stroke between the two interventions remains inconclusive, with results varying across different investigations [78,79,80].

Both revascularization strategies have been extensively evaluated in the management of patients with ischemic heart failure. Most of the evidence supports CABG, demonstrating its association with improved long-term survival [81,82,83].

Nevertheless, the number of studies investigating this issue in patients with both diabetes and heart failure is limited. Therefore, the findings of these studies may have a profound impact on clinical decision-making. One such study is a propensity-matched analysis conducted by Nagendran et al. [84] from 2004 to 2016, which included 1738 patients. The patients were stratified into two groups based on their LVEF: one group with an LVEF of 35–49%, and another with an EF < 35%. The mean follow-up duration was 5.5 years. The results indicated that PCI was associated with a higher risk of MACCEs, defined as the composite of death, stroke, MI, and repeat revascularization, and the mortality rate compared to CABG in both cohorts. The risk of myocardial infarction was comparable in patients with an EF of 35–49%, while PCI was associated with an elevated risk of myocardial infarction in patients with an EF < 35%. Stroke rates did not show significant differences between the two revascularization approaches, irrespective of EF [84].

**Table 1:** The most relevant RCTCs comparing CABG, PCI, and OMT.

Study Name	STICH/STICHES		REVIVED-BCIS2		FREEDOM		CARDia		BARI		SYNTAX		COURAGE		ISCHEMIA	
Year of first published results	2011 [49]/2016 [51]		2022 [53]		2012 [55]		2010 [62]		1996 [64]		2009 [68]		2007 [73]		2020 [74]	
Primary outcome	Death from any cause		Death from any cause or hospitalization for HF		MACCE *		MACCE **		All-cause mortality (1); Q-wave-myocardial-infarction-free survival rate (2)		MACCE ***		Death from any cause or nonfatal myocardial infarction		Death from cardiovascular causes, myocardial infarction, or hospitalization for unstable angina, heart failure, or resuscitated cardiac arrest	
Number of participants	1212		700		1900		600		1829		1800		2287		5179	
Population	CAD + HF		CAD + HF		MVCAD + DM		CAD + DM		MVCAD		Three-vessel and/or left main CAD		Stable CAD		Stable CAD	
Treatment modality	CABG + OMT	OMT	PCI + OMT	OMT	CABG	PCI	CABG	PCI	CABG	PCI/POBA	CABG	PCI	PCI + OMT	OMT	CABG/PCI + OMT	OMT
Severe LV dysfunction (%)	100	100	100	100	1.7	3.3	16.4	20.3	Unknown	Unknown	1.3	2.5	Exclusion criterion		Exclusion criterion	
DM (%)	39	40	39	43	100	100	100	100	25/20 on treatment	24/19 on treatment	24.6	25.6	32	35	41.4	42.2
Primary outcome occurrence (%)	58.9	66.1	37.2	38.0	18.7	26.6	10.5	13.0	10.7 (1); 80.4 (2)	13.7 (1); 78.7 (2)	26.9	37.3	19.0	18.5	12.3	13.6
*p* value	0.12 (5-yYear FU) [49]/0.02 (10-yYear FU) [51]		0.96 (2-yYear FU) [53]		0.005 (5-yYear FU) [55]		0.393 (1-yYear FU) [62]		0.19 (1); 0.84 (2) (5-yYear FU) [64]		<0.0001 (5-yYear FU) [70]		0.62 (4.6-yYear FU) [73]		0.34 (3.2-yYear FU) [74]	

* Death from any cause, myocardial infarction, or stroke; ** dDeath, nonfatal myocardial infarction, or nonfatal stroke; *** dDeath from any cause, stroke, myocardial infarction, or repeat revascularization; RTC—randomized controlled trial; CABG—coronary artery bypass graft; PCI—percutaneous coronary intervention; OMT—optimal medical therapy; LV—left ventricle; MACCE—major adverse cardiac and cerebrovascular events; CAD—coronary artery disease; MVCAD—multivessel coronary artery disease; DM—diabetes mellitus; POBA—plain old balloon angioplasty; FU—follow- up.

## 8. Special Populations

### 8.1. Advanced Age

Recent studies have provided significant insights into the relationship between aging and CAD, highlighting the complex interplay between vascular aging, disease progression, and therapeutic strategies. Aging impairs endothelial function, causing a loss of elasticity in arterial walls. This leads to increased arterial stiffness, thickening of the intimal and medial layers, and narrowing of the lumen, which restricts blood flow and raises the risk of ischemic events. These changes significantly contribute to the development of cardiovascular diseases, including CAD, heart failure, and stroke [85]. A 2023 study, analyzing electronic health records data from the National Health and Nutrition Examination Survey (NHANES), revealed that patients with diabetes and stable CAD tend to be older and have higher multimorbidity. This category is at an increased risk of developing major cardiovascular events, suggesting that age is a significant factor in the prognosis of diabetic patients with CAD [86]. Elderly patients with newly diagnosed diabetes undergoing PCI exhibit worse long-term outcomes, including higher rates of major adverse cardiovascular events (MACEs) and mortality compared to those without diabetes, emphasizing the need for vigilant screening and tailored therapeutic approaches in this population [87]. Moreover, older diabetic patients who undergo PCI exhibit higher rates of target vessel failure and repeat revascularization compared to non-diabetic counterparts. The increased risk is primarily attributed to altered inflammatory pathways and endothelial dysfunction [88].

### 8.2. Left Main Coronary Artery Disease

LMCAD and diabetes are linked to significant cardiovascular complications and an increased risk of mortality [89]. A recent meta-analysis evaluated outcomes of patients with LMCAD, comparing two revascularization strategies, CABG and PCI, in those with and without diabetes [90]. Data were pooled from four major RCTs (the SYNTAX trial [64], the PRECOMBAT [Premier of Randomized Comparison of Bypass Surgery Versus Angioplasty Using Sirolimus-Eluting Stent in Patients With Left Main Coronary Artery Disease] trial [91], the NOBLE [Nordic–Baltic–British Left Main Revascularisation Study] trial [92], and the EXCEL [Evaluation of XIENCE Versus Coronary Artery Bypass Surgery for Effectiveness of Left Main Revascularization] trial [93]). The primary findings of this meta-analysis indicated that diabetes was linked to higher rates of mortality and cardiovascular events over five years, irrespective of the revascularization method used. While the risk of death was comparable between CABG and PCI, PCI was associated with a lower incidence of early stroke regardless of diabetes status. However, PCI was linked to an increased risk of spontaneous myocardial infarction and repeat revascularization, with these risks accumulating more substantially over time in patients with diabetes [90]. The 2024 ESC/EACTS Guidelines for the management of chronic coronary syndromes recommend the LMCAD revascularization strategy based on the disease’s complexity and individual patient characteristics, emphasizing the importance of a multidisciplinary approach. CABG is recommended as the preferred revascularization strategy in patients with LMCAD and complex coronary anatomy (SYNTAX score > 32), particularly when associated with multivessel disease. CABG provides more complete revascularization, improved long-term survival, and reduced rates of MACCEs compared to PCI in this patient group. On the other hand, PCI is recommended as an alternative to CABG in patients with LMCAD and low to intermediate anatomical complexity (SYNTAX score ≤ 32) and are deemed unsuitable for surgery or prefer PCI [57]. Advancements in stent technology and procedural techniques have demonstrated equivalent long-term outcomes to CABG in selected patients with LMCAD and lower anatomical complexity, as shown in the EXCEL and NOBLE trials [92,93]. However, in high-risk patients with diabetes, CABG is preferred over PCI due to superior outcomes in long-term survival and reduction of MACCEs [54]. In stable patients with LMCAD, the 2023 ACC/AHA/SCAI guidelines also prioritize CABG as the preferred revascularization strategy to improve survival [59]. A recent study found that PCI is associated with a greater risk of all-cause death and repeat revascularization in nonelderly patients compared with CABG in patients with LMCAD. Among patients over 75 years, PCI and CABG resulted in similar risks of all-cause death and repeat revascularization [94]. In conclusion, age and diabetes are crucial in determining outcomes in LMCAD. Younger patients benefit more from CABG, while older patients experience similar outcomes with CABG and PCI. Diabetic patients, however, show superior long-term results with CABG, highlighting the importance of individualized treatment strategies.

### 8.3. Chronic Kidney Disease

CKD remains a significant independent risk factor for atherosclerosis [95]. The accelerated atherosclerotic process in CKD is driven by a complex interplay of factors, including persistent systemic inflammation, enhanced ROS production, dysregulated lipid and electrolyte homeostasis, mitochondrial dysfunction, DNA damage, and the accumulation of uremic toxins [96]. Currently, about 40% of patients with T2DM are affected by CKD, and this figure is expected to rise [97]. The recent ISCHEMIA-CKD (International Study of Comparative Health Effectiveness with Medical and Invasive Approaches–Chronic Kidney Disease) trial investigated patients with advanced CKD (eGFR < 30 mL/min/1.73 m² or on dialysis) and moderate-to-severe myocardial ischemia identified by stress testing. The results indicated that an invasive strategy, incorporating coronary angiography and PCI, did not outperform conservative medical management in reducing the incidence of death or non-fatal myocardial infarction. These findings challenge the presumed benefits of invasive interventions in this population, highlighting the importance of individualized treatment approaches and the risks inherent in advanced CKD [98]. A comprehensive pooled analysis demonstrated that the presence of CKD was strongly correlated with a substantially elevated risk of both early and late all-cause mortality compared to individuals without CKD. Interestingly, no significant difference was found in the incidence of revascularization procedures between patients with CKD and those without [99]. According to the latest 2024 ESC/EACTS Guidelines for the management of chronic coronary syndromes, the preferred revascularization strategy for patients with CKD and LMCAD is CABG, considering the higher rates of restenosis and repeat revascularization associated with PCI [54]. A recent study that evaluated coronary revascularization strategies in patients with CKD and diabetes showed that CABG provides a significant survival advantage over PCI, particularly for those with multivessel disease. Long-term benefits of CABG in diabetic patients are attributed to its ability to bypass diffuse atherosclerosis, a common feature in diabetes, whereas PCI primarily targets focal lesions [100]. Over the last decades, evidence suggests that CABG provides a superior long-term survival benefit compared to PCI in patients with end-stage renal disease requiring dialysis [101].

### 8.4. Peripheral Artery Disease

Among patients with CAD, 7–16% are diagnosed with lower extremity artery disease, which is often asymptomatic and masked by cardiac symptoms, but significantly worsens prognosis [102]. According to the latest 2024 ESC/EACTS Guidelines for the management of chronic coronary syndromes, which considered myocardial revascularization strategies, the decision between CABG and PCI in patients with PAD remains a subject of debate, particularly in the absence of robust evidence. In such cases, a multidisciplinary heart team approach is essential to ensure individualized, patient-centered decision-making [57]. The recent INCORPORATE (Intentional Coronary Revascularization Versus Conservative Therapy in Patients Undergoing Peripheral Artery Revascularization Due to Critical Limb Ischemia) trial investigated the effects of routine coronary angiography followed by ischemia-guided revascularization compared to medical therapy alone in patients undergoing peripheral revascularization due to critical limb ischemia. The study aimed to determine which approach better reduced the composite outcome of all-cause mortality and spontaneous myocardial infarction. Ultimately, the findings did not demonstrate the superiority of the invasive strategy over conservative treatment in reducing primary outcomes in this high-risk population. Furthermore, the results were similar when patients were stratified by diabetes status, indicating that diabetes did not influence the efficacy of the interventions [103].

### 8.5. Chronic Obstructive Pulmonary Disease

Recent studies highlight COPD as a key independent risk factor for adverse outcomes after revascularization. A 2023 analysis revealed that individuals with COPD are more likely to suffer from cardiovascular conditions such as coronary heart disease, heart failure, myocardial infarction, and diabetes compared to those without COPD [104]. COPD significantly impacts long-term outcomes in patients with diabetes and multivessel CAD undergoing revascularization. While CABG demonstrates superior survival benefits over PCI in this population, the presence of COPD increases perioperative risks and adverse events. Optimal revascularization strategies for COPD patients require careful risk stratification and multidisciplinary management including pulmonary optimization, precise revascularization strategy selection, and robust perioperative care [79].

## 9. Conclusions and Future Directions

The role of myocardial revascularization in patients with diabetes and heart failure remains an area of ongoing clinical debate, primarily due to the lack of high-quality, conclusive evidence. In the absence of definitive RCTs, an individualized, patient-centered approach is essential. This approach should consider the complexity of CAD, the patient’s overall health status, and the presence of comorbidities, ensuring that treatment is tailored to meet each individual’s needs.

Observational studies have suggested that CABG may offer superior outcomes compared to PCI, particularly for patients with multivessel disease, LMCAD, or complex coronary anatomy. CABG has been associated with better long-term survival, a reduced need for recurrent revascularization procedures, and improved functional outcomes, including better symptomatic relief and fewer hospitalizations due to heart failure exacerbations [90]. However, advancements in PCI techniques, including improved stent technology and refined procedural strategies, have led to enhanced post-procedural outcomes [105].

In patients with HFrEF and stable ischemic heart disease, myocardial revascularization should be considered when symptoms such as recurrent angina or a positive stress test persist despite optimal medical therapy. For individuals with single-vessel disease, PCI is often the most appropriate option. However, for those with multivessel disease, LMCAD, or concomitant diabetes, a heart team decision is necessary to assess the risks and benefits of CABG, which remains the preferred treatment choice in many cases [106].

The ongoing STICH3C trial (NCT05427370), which is currently in the patient recruitment phase, holds promise for providing valuable insights into this critical issue. This well-designed, large-scale trial aims to compare the efficacy of CABG versus PCI specifically in patients with ischemic cardiomyopathy, potentially addressing many unanswered questions and offering much-needed evidence to refine management strategies for this complex patient population [107].

In conclusion, while observational studies provide some guidance, rigorous RCTs are urgently needed to refine our understanding and management of myocardial revascularization in patients with diabetes and heart failure. As we await the results of the STICH3C trial and other ongoing research, a multidisciplinary approach that takes into account the patient’s coronary anatomy, comorbidities, and individual health status remains essential for determining the optimal treatment strategy.

## Data Availability

No new data were created or analyzed in this study. Data sharing is not applicable to this article.
